# Effects of Inflammatory Disease on Clinical Progression and Treatment of Ischiogluteal Bursitis: A Retrospective Observational Study

**DOI:** 10.5704/MOJ.2011.007

**Published:** 2020-11

**Authors:** YH Roh, SJ Yoo, YH Choi, HC Yang, KW Nam

**Affiliations:** Department of Orthopaedics, Jeju National University Hospital, Jeju City, Republic of Korea

**Keywords:** ischiogluteal bursitis, clinical course, inflammatory disease, treatment responsiveness, risk factor

## Abstract

**Introduction:**

The symptoms of Ischiogluteal Bursitis (IGB) are often nonspecific and atypical, and its diagnosis is more challenging. Moreover, it is difficult to predict cases of chronic progression or poor treatment response. Therefore, the aim of this study was to investigate the clinical course of IGB patients and identify factors that are predictive of failure of conservative treatment.

**Materials and Methods:**

Our study consisted of IGB patients diagnosed between 2010 March and 2016 December who had been followed-up for at least one year. Structured questionnaires and medical records were reviewed to analyse demographic characteristics, lifestyle patterns, blood tests, and imaging studies. We categorized the cases into two groups based on the response to conservative treatment and the need for surgical intervention.

**Results:**

The most common initial chief symptoms were buttock pains in 24 patients (37.5%). Physical examinations showed the tenderness of ischial tuberosity area in 59 (92.2%) patients, but no specific findings were confirmed in 5 patients (7.8%). 51 patients (79.7%) responded well to the conservative management, 11 patients (17.2%) needed injection, and 2 patients (3.1%) had surgical treatment performed due to continuous recurrence. There was no difference in demographic and blood lab data between the two groups. However, the incidence of inflammatory diseases (response group: 10.3% vs non-response group: 66.7%, p=0.004) was significantly different between the two groups.

**Conclusion:**

The diagnosis of IGB can be missed due to variations in clinical symptoms, and cautions should be exercised in patients with inflammatory diseases as conservative treatment is less effective in them, leading to chronic progression of IGB.

## Introduction

Pain in the gluteal region may be due to a number of causes including sciatica, lumbar disc degeneration, piriformis syndrome, sacroiliitis, and hip bursitis^1,2^. More than 140 bursal sacs have been described in the human body, any of which can be involved in the disease. Eighteen bursa sacs are located around the hip joint. Of those hip bursa sacs, trochanteric, ischiogluteal, and iliopsoas bursa are often problematic^3^. The ischiogluteal bursa is an adventitial bursa located between the ischial tuberosity and the gluteus maximus muscle, smoothing movement by reducing friction between the two^3,4^. However, the bursa may be irritated and become inflamed by sitting on a hard surface for a prolonged period.

Ischiogluteal bursitis (IGB), previously known to be “weaver’s bottom”, is an uncommon disorder nowadays, and it is often overlooked as a cause of buttock pain^2,5^. As the bursa lies in a close contact with the sciatic and posterior femoral cutaneous nerve, IGB can mimic the symptoms of radiculopathy. Enlargement of this bursa could clinically mimic a soft tissue neoplasm^6-8^. In addition, the symptoms of IGB are varied and often non-specific, making it difficult to diagnose and confused with many other diseases. Moreover, it is difficult to predict cases of chronic progression or poor treatment response.

There has been currently little literature on IGB, and there are no reports directly looking into the clinical course of the disease. Therefore, the aim of this study is to investigate the clinical course of IGB patients and identify factors that are predictive of failure of conservative treatment.

## Materials and Methods

This study is a part of a retrospective observational cohort study of IGB. We screened patients who were diagnosed with IGB at the Jeju National University Hospital between March 2010 and December 2016. Within the retrospective cohort, patients who were diagnosed IGB and received outpatient and inpatient treatment were considered eligible for the study. Patients who were lost in follow-up for more than a one year and did not respond to the interview were excluded.

In most cases, the first diagnosis of IGB was sufficient through clinical symptom and physical examination. The patients were finally diagnosed by confirming the enlargement of the bursa by bedside ultrasonography. If the diagnosis was unclear, the patients were referred to radiologists for detailed ultrasound and magnetic resonance imaging (MRI).

Clinical symptoms, medical history findings, blood lab tests, and medical imaging were retrospectively reviewed. Imaging such as plain radiography, ultrasonography and MRI findings were confirmed by musculoskeletal radiologists at our institution. The symptom progressions of the IGB and the most recent outcome were confirmed through telephone interviews. The Jeju National University Hospital institutional review board approved this retrospective cohort study.

We designed a structured questionnaire on the IGB characteristics. The survey was conducted on the first symptom characteristics, duration of symptom, findings on physical examinations, treatment methods, time to sit per day, whether sitting on the floor or on a chair, type and strength of occupation, and history of trauma to the affected buttock area before three months of outpatient visit. To evaluate treatment effectiveness and sustainability, a visual analogue scale (VAS) was used to compare the pain level between the initial and last visit.

Demographic characteristics used in the analysis included gender, age, body mass index (BMI), and identified inflammatory disease conditions that could affect the IGB. In addition, the medical records were reviewed to analyse inflammatory factors including erythrocyte sedimentation rate (ESR), and C-reactive protein (CRP), coagulation factors such as platelets, prothrombin time international normalized ratio (PT INR), and activated partial thromboplastin time (aPTT), and liver function and kidney function levels.

In this study, if the pain VAS did not improve by more than 50% and persisted for more than six months despite conservative and injection treatment, and when surgical treatment was required, the patient was defined as 'nonresponse IGB'. Conversely, when symptoms improved by conservative and injection treatment, the patient was defined as a ‘response IGB’^9^.

Treatment for IGB patients proceeded in three phases. First phase was the level of conservative treatment, involving education on avoidance therapy to minimize repetitive trauma, a medication that could control symptoms such as non-steroidal anti-inflammatory drugs (NSAIDs), and physical therapy using extracorporeal shockwave therapy (ESWT). The second phase was to inject a mixture of steroid (triamcinolone 40mg) and local anesthetic (ropivacaine 150mg) into the ischiogluteal bursa for a more direct effect. When treatment trials in the previous two phases failed, the operation was performed in the final phase. All the interviews were performed by one orthopaedic surgeon (RYH) using this questionnaire.

All the descriptive data are presented as a number (percentage) or mean ± standard deviations unless otherwise specified. For the group comparisons, categorical variables were analysed with Chi-square tests or Fisher’s exact tests, while continuous variables were compared with Student’s t-tests or Mann-Whitney tests according to the normal distribution of the variable. For categorical variables with three or more independent variables, the linear by linear association method was used. A multiple logistic regression analysis was performed to identify independent risk factors for non-response IGB. Statistical analyses were performed using SPSS statistical software version 20 [IBM® Corp, Armonk, NY, USA]. A P-value < 0.05 was considered statistically significant.

## Results

**Fig. 1:**
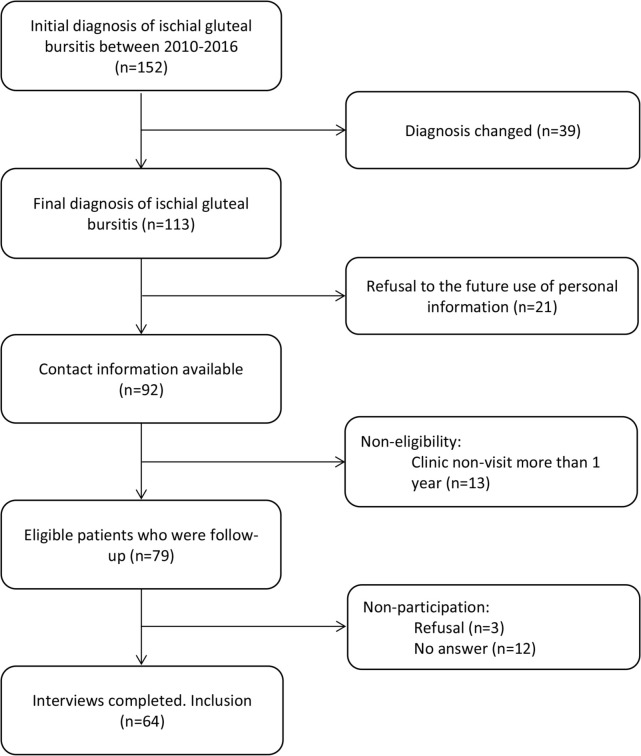
Patient enrollment flow chart. This study involved ischiogluteal bursitis in 64 patients.

The study flow chart is shown in (Fig. 1). A total of 64 patients (current age, mean 65.8 years; range 45-87) were included in the final analysis. The mean time of follow-up period was 5.6 years (range, 2.1-9.4). The demographics and baseline characteristics are summarized in (Table I). There were more women (54.7%) in the analysis. Ages of the female patients varied from 45 to 87 (mean 67.3) years. The male accounted for 45.3% and were aged from 19 to 86 (mean 66.8) years. The average BMI of patients was measured at 24.0 kg/m^2^ (range 15.2-29.9).

**Table I:** Baseline characteristics included in this study [% (n)] or [Mean ± SD (n)]

Variables	Total patients (n=64)
Gender	
Female	54.7 (35)
Male	45.3 (29)
Age (year)	67.8 ± 11.3
Height (cm)	159.2 ± 96.5
Weight (kg)	61.1 ± 12.5
BMI^a^ (kg/m^2^)	24.0 ± 3.4
Occupation	
Physical Job	29.7 (19)
Office Job	42.2 (27)
None	28.1 (18)
Trauma history on buttock	
Yes	35.9 (23)
No	64.1 (41)
Affected Side	
Left	40.6 (26)
Right	59.4 (38)
Sitting lifestyle	
Sitting on chair	65.6 (42)
Sitting on floor	34.4 (22)
Sitting time	
< 4 hours	62.5 (40)
≥ 4 hours	37.5 (24)
Inflammatory disease	15.6 (10)
Rheumatoid arthritis	7.8 (5)
Gout	4.7 (3)
Pseudogout	1.6 (1)
Skin infection	1.6 (1)
Disease duration (month)	5.4 ± 5.2
Follow-up duration (year)	5.6 ± 2.1
Blood test	
Hemoglobin (g/dL)	13.0 ± 1.8
Platelet (x10^3^/ μL)	224.1 ± 58.7
WBCb (x10^3^/ μL)	6.8 ± 2.1
Neutrophil (mm^3^)	4.4 ± 1.8
ESR^c^ (mm/h)	20.5 ± 15.4
CRP^d^ (mg/dL)	1.1 ± 1.5
Uric acid (mg/dL)	6.1 ± 2.2
FBS^e^ (mg/dL)	109.6 ± 29.9
AST^f^ (U/L)	26.9 ± 9.4
ALT^g^ (U/L)	24.1 ± 11.1
BUN^h^ (mg/dL)	18.8 ± 8.7
Creatinine (mg/dL)	1.0 ± 0.6
PT INR^i^ (second)	1.0 ± 0.1
aPTT^j^ (second)	28.9 ± 4.9
VAS^k^ score	
Initial	4.5 ± 1.2
Last follow-up	1.8 ± 0.8

a: Body mass index, b: White blood cell, c: Erythrocyte sedimentation rate, d: C-reactive protein, e: Fasting blood sugar, f: Aspartate aminotransferase, g: Alanine aminotransferase, h: Blood urea nitrogen, i: Prothrombin time and international normalized ratio, j: Activated partial thromboplastin time, k: Visual analog scale

As for the current occupational status of patients, 19 patients (29.7%) engaged in physical labor such as agriculture, fishing, and construction, while 27 patients (42.2%) were engaged in office workers such as public officers. In addition, there were 18 patients (28.1%) who did not have current occupational status. The laterality of affected buttock is more dominant in the right side (59.4%). There were 23 patients who had a history of previous trauma on the affected buttocks, accounting for 35.9 percent. The most common mode of trauma was fall-down, and there was only one case of minor traffic accident. There were five patients with a history of treatment for rheumatoid arthritis, three patients with gout, and one patient with pseudo-gout. There also was one patient who had been treated for infectious skin disease on the affected buttock area.

The initial chief symptoms varied. The most common chief complaint was buttock pain, observed in 24 patients (37.5%). There were 17 patients (26.5%) who complained of an inguinal pain. There were also 9 patients (14.1%) who complained of a radiating pain to the lower extremity of the ipsilateral side. There were 8 patients (12.5%) who complained of thigh pain, and 5 patients (7.8%) complained of discomfort with buttock mass without pain. In addition, one patient (1.6%) complained of coccyx area pain (Table II).

**Table II:** Clinical Features at the Initial chief presentation [% (n)]

Initial Symptoms	Ratio of Cases % (n)
Buttock pain	37.5 (24)
Inguinal pain	26.5 (17)
Radiating pain	14.1 (9)
Thigh pain	12.5 (8)
Buttock mass	7.8 (5)
Coccyx area pain	1.6 (1)

When IGB was diagnosed for the first time, there were also a variety of positive findings from physical examination. The tenderness of ischial tuberosity area was identified in 59 (92.2%) patients. In 14 (21.9%) patients, ipsilateral lower extremity radiating pain and a positive straight leg raise test were observed. There were 9 (14.1%) patients who complained of pain or had severe pain in the flexion-adduction-internal rotation test, which puts the hip joint to flexed 90°, adducted and internally rotated. Seven (10.9%) patients were positive for Patrick's test, which puts the hip joint to flexed 90°, abducted and externally rotated in a different direction. There were also five patients (7.8%) who did not show any specific findings on the physical examination (Table III).

**Table III:** Physical Examination [% (n)]

Initial findings	Ratio of Cases % (n)
Tenderness on ITa	92.2 (59)
SLRb: positive	21.9 (14)
FADIRc test: positive	14.1 (9)
Patrick's test: positive	10.9 (7)
Non-specific finding	7.8 (5)

a: Ischial Tuberosity; b: straight leg raise; c: flexion-adduction-internal rotation

Most of the patients diagnosed with IGB responded well to the conservative treatment, improving symptoms in 51 patients (79.7%). Conservative treatment alone was insufficient for 11 patients (17.2%), and an intra-bursal injection was performed. Fifty-eight patients out of 64 patients responded well to treatment, and in 6 patients were non-response IGBs who persisted in symptoms and did not respond to conservative treatment. Two of patients (3.1%) with unsatisfactory conservative treatment had their bursa excised by surgical intervention, while four other patients with persistent symptoms despite conservative measures continued to receive treatment.

Sixty-four patients were analysed in the two groups. The non-response IGB group included six patients (9.4%) who had persistent clinical symptoms and needed surgical treatment. The ‘response IGB’ groups consisted of 58 patients (90.6%) whose symptoms improved with conservative treatment. Univariate analysis was used to assess the effect of variables between the two groups with different treatment responsiveness. No statistically significant differences were observed with respect to gender (p=0.209), age (p=0.325), BMI (p=0.955), occupation (p=0.588), trauma history (p=0.632), affected side (p=0.680), sitting lifestyle (p=0.170), sitting time (p=0.664), ESR (p=0.098), CRP (p=0.101), uric acid (p=0.711), and other blood test values. However, statistically significant differences were observed for presence of inflammatory diseases (p=0.004) (Table IV).

**Table IV:** Univariate comparison between response and non-response groups [% (n)] or [Mean ± SD (n)]

Variables	Total patients (n=64)	Non-response group (n=6)	P value
Gender			0.209
Female	51.7 (30)	83.3 (5)	
Male	48.3 (28)	16.7 (1)	
Age (year)	68.3 ± 11.2	63.0 ± 12.3	0.325
Height (cm)	159.8 ± 9.7	153.0 ± 7.0	0.066
Weight (kg)	61.6 ± 12.9	57.0 ± 8.7	0.551
BMI^a^ (kg/m^2^)	24.0 ± 3.4	24.7 ± 4.2	0.955
Occupation			0.588
Physical Job	29.3 (17)	33.3 (2)	
Office Job	44.8 (25)	33.3 (2)	
None	25.9 (16)	33.3 (2)	
Trauma history on buttock			0.632
Yes	22.4 (21)	33.3 (2)	
No	77.6 (37)	66.7 (4)	
Affected Side			0.680
Left	39.7 (23)	50.3 (3)	
Right	60.3 (35)	50.3 (3)	
Sitting lifestyle			0.170
Sitting on chair	67.2 (40)	33.3 (2)	
Sitting on floor	32.8 (18)	66.7 (4)	
Sitting time			0.664
< 4 hours	63.8 (37)	33.3 (3)	
≥ 4 hours	36.2 (21)	66.7 (3)	
Inflammatory disease	10.3 (6)	66.7 (4)	0.004
Rheumatoid arthritis	5.2 (3)	33.3 (2)	
Gout	3.4 (2)	16.7 (1)	
Pseudogout	1.7 (1)	0.0 (0)	
Skin infection	0.0 (0)	16.7 (1)	
Disease duration (month)	4.5 ± 2.7	14.3 ± 12.3	0.050
Follow-up duration (year)	5.6 ± 2.1	5.4 ± 2.4	0.694
Blood test			
Hemoglobin (g/dL)	13.1 ± 1.8	12.3 ± 2.2	0.450
Platelet (x10^3^/ μL)	225.1 ± 60.0	214.0 ± 47.2	0.551
WBC^b^ (x10^3^/ μL)	6.9 ± 2.1	6.0 ± 1.0	0.073
Neutrophil (mm3)	4.4 ± 1.9	3.8 ± 0.6	0.093
ESR^c^ (mm/h)	19.9 ± 15.9	26.3 ± 7.6	0.098
CRP^d^ (mg/dL)	1.1 ± 1.6	0.9 ± 0.3	0.101
Uric acid (mg/dL)	6.2 ± 2.2	6.1 ± 1.9	0.711
FBS^e^ (mg/dL)	110.2 ± 31.3	104.2 ± 6.0	0.314
AST^f^ (U/L)	27.0 ± 9.8	25.5 ± 2.9	0.937
ALT^g^ (U/L)	23.5 ± 11.1	29.8 ± 10.4	0.233
BUN^h^ (mg/dL)	18.9 ± 9.1	18.4 ± 4.5	0.217
Creatinine (mg/dL)	1.0 ± 0.6	0.8 ± 0.1	0.902
PT INR^i^ (second)	1.0 ± 0.1	1.1 ± 0.1	0.987
aPTT^j^ (second)	29.2 ± 5.0	25.8 ± 2.1	0.112
VAS^k^ score			
Initial	4.4 ± 1.2	5.0 ± 0.9	0.256
Last follow-up	1.7 ± 0.7	3.3 ± 0.5	0.000

a: Body mass index, b: White blood cell, c: Erythrocyte sedimentation rate, d: C-reactive protein, e: Fasting blood sugar, f: Aspartate aminotransferase, g: Alanine aminotransferase, h: Blood urea nitrogen, i: Prothrombin time and international normalized ratio, j: Activated partial thromboplastin time, k: Visual analog scale

Multiple logistic regression analyses were performed to identify independent factors associated with non-response IGBs. As a result, it was found that inflammatory disease is an independent risk factor for non-response IGB (Odds ratio: 40.380; 95% Confidence interval: 2.304-707.719; p=0.011) (Table V).

**Table V:** Logistic regression analysis of non-response group

Variables	B value	95% CI^a^	P value
Age	0.950	0.846-1.066	0.384
BMI^b^	1.157	0.762-1.757	0.493
Trauma history on buttock	2.472	0.167-36.683	0.511
Sitting lifestyle	8.907	0.640-102.492	0.106
Sitting time	1.823	0.142-23.434	0.645
Inflammatory disease	40.380	2.304-707.719	0.011
WBC^c^	0.999	0.998-1.000	0.194
ESR^d^	1.002	0.930-1.080	0.959
CRP^e^	1.408	0.435-4.560	0.568

a: Confidence interval, b: Body mass index, c: White blood cell, d: Erythrocyte sedimentation rate, e: C-reactive protein

## Discussion

In general, bursitis is encountered equally in the male and female populations, reported across all ages. However, some types of bursitis have documented a female predilection, specifically pes anserine and trochanteric bursitis. Furthermore, these forms of bursitis are more common in obese individuals^10,11^. On the contrary, men are more often affected by olecranon bursitis. This is presumed to be due to differences in occupational characteristics between men and women, with an increased rate of men performing manual labour for a living^12^. In this study, more IGB occurred in women (54.7%) than in men (45.3%). BMI among patients with IGB varied from 15.2 to 29.9 kg/m^2^. Occupational variables have been observed at a higher prevalence in sedentary jobs (42.2%) than physically demanding labour (29.7%), but many IGBs have occurred even in non-working group (28.1%). These results are consistent with the previous idea that IGB is mainly due to the chronic and sustained irritation of bursa and occurs most often in people living a sedentary life^13,14^.

IGB may occur in various conditions, much like many other bursal inflammations. Autoimmune diseases such as rheumatoid arthritis, systemic lupus erythematosus, and scleroderma have been reported to cause bursitis. Bursa inflammation can also be caused by uremia seen in gout and chronic kidney disease. Ischial gluteal bursa is a deep bursa, meaning that it is less susceptible to the contiguous spread of infectious organisms. Although rare, infectious ischial bursitis associated with septicemia and septic arthritis^15,16^. However, previous studies have not provided enough evidence to conclude that these conditions are related to IGB.

Chronic microtrauma, presented as one of other causes for IGB, is also common in other superficial bursitis. Microtrauma results from chronic repetitive friction on the tissue overlying the bursa and its underlying bony prominence^17,18^. In this study, a total of 23 patients (35.9%) were identified with a previous history of trauma, which have contributed to affect IGB.

IGB patients often complain of a variety of clinical symptoms. Patients may present with gluteal pain and/or upper posterior thigh radiating pain following prolonged sitting or exercise^19^. Patients with IGB most commonly complain of low grade, pinpoint, aching pain that is worsened by sitting down or stretching the gluteus maximus muscle^20^. Patients may complain of problems with sleeping due to the pain. Patients also may have reduced mobility and swelling associated with this condition. In this study, we identified the most common chief complains of the first outpatient visit. Buttock pain was the most frequent chief complaint, reported in 24 patients (37.5%), whereas other 40 patients (62.5%) complained of other symptoms. It is important to note that various clinical symptoms can occur, and detailed history taking should be performed.

On physical examination, tenderness over the buttock was most notable. Patients tend to address pain in passive flexion of the hip joints and difficulty in extension of hip^20^. The patient may feel pain with stretching^5^. A soft tissue mass, which tends to be well-defined, non-mobile, and slightly tender, may be present in the gluteal region of the affected hip^21^. When erythema overlying the gluteal regions is a major clinical presentation, infectious causes should be suspected. In this study, tenderness on ischial tuberosity was positive in 59 patients (92.2%) during a physical examination, while no specific findings were confirmed in the 5 patients (7.8%). Therefore, even if there is no tenderness of ischial tuberosity in physical examination, IGB should not be completely excluded.

**Fig. 2:**
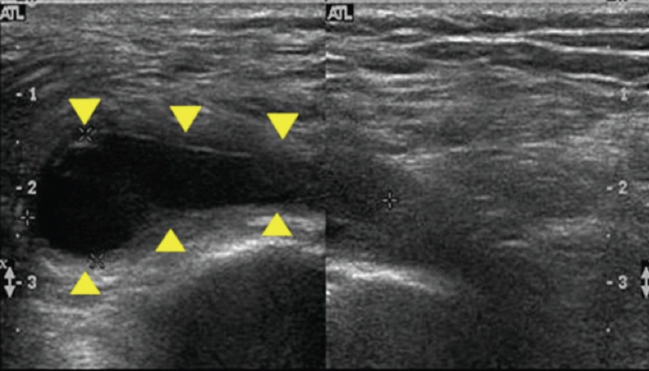
The image of ultra-sonography in a 76-year female shows hypoechoic lesion (arrow head) with acoustic enhancement represents the ischiogluteal bursitis.

**Fig. 3:**
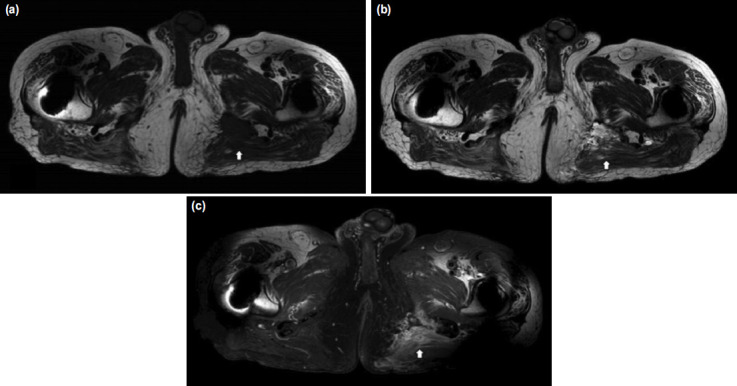
The axial image of the pelvis magnetic resonance imaging in a 79-year old male. (a) A cystic lesion (arrow) showing low signal intensity was observed in T1-weighted view. (b) It was observed that the same lesion (arrow) showed high signal intensity in T2-weighted view. (c) In the contrast-enhanced T1-weighted view, it is observed that the wall of the cystic lesion (arrow) is enhanced.

IGB requires a detailed history taking, and most diagnosis can be made by physical examination and clinical symptoms. When there is a palpable mass over ischial tuberosity with tenderness, ultrasonography is a useful tool in detection of is location as well as in further examination such as aspiration (Fig. 2). MRI is the most sensitive investigation tool for differential diagnosis of bursitis but less cost-effective in most of the time, unless malignancy is suspected in patients with a palpable mass over the gluteal region^13,15,22,23^. T1-weighted scans show an injury with intermediate intensity. T2-weighted scans show a higher intensity of this lesion, suggesting a fluid-filled space (Fig. 3).

Treatment of IGB focuses on symptom control. Primary treatment in IGB is lifestyle modification by avoiding physical activities or daily habits that have caused the symptoms in the first place^24,25^. In addition, the use of ESWT in conservative management is recommended to be beneficial in reducing adhesion in chronic bursitis by breaking down scar tissues, increasing extensibility, mobility of surrounding structure, and ultimately promoting normal orientation of collagen fibers^26,27^. NSAIDs are also beneficial in decreasing inflammation and pain. An intra-bursal injection of corticosteroid can be considered in cases of unbearable and unrelenting pain^1,12,15^. In this study, most patients responded well to medication treatment using NSAIDs. There were only 11 patients (17.2%) who needed intra-bursal injection treatment.

Surgical treatment is recommended in patients with persistent or repeated superficial bursitis and significant enlargement of bursa causing functional disability. Surgical treatment includes open or endoscopic bursectomy and partial excision of the underlying bony tissue^17^. In this study, open bursectomy was performed on two patients (3.15%), each of whom had a history of gout and skin infection. Two cases that initially presented as aseptic IGB developed into septic IGB with erythema, which required antibiotics therapy for several months after aspiration. However, the medication failed and symptoms persisted, eventually leading to open surgical intervention.

Patients diagnosed with IGB tend to remain asymptomatic within weeks to months not requiring any certain treatment^28^. However, in rare cases it persists chronically and does not respond to typical conservative treatment. In this study, comparative analysis was conducted between those who responded well to the treatment and those who did not. Occupation, trauma history, sitting lifestyle and time, and obesity, commonly considered causal in IGB, could not be observed for statistical significance with non-response IGB^29,30^. Patients with underlying inflammatory diseases, such as rheumatoid arthritis, gout, and skin infection, showed less responsiveness to conservative management, and two of the patients in our study required open surgical intervention due to failure of medical treatment^9,31^. Therefore, underlying inflammatory diseases in IGB patients is an important factor to consider in treatment response and patient education.

There are several limitations to this study. One of the limitations includes its retrospective design and small size of the cases with non-response IGB. Another limitation is the ability to present accurate information because some interviews are retrospective. On the other hand, the most notable strength of our study is that all patients were treated in the same way by one skilled orthopaedic surgeon in the same institution. It also presents the possibility of incorporating into future studies by reporting areas that were not noted in previous studies.

## Conclusion

IGB is often overlooked because it is diagnosed clinically and responds well to conservative treatment. However, the diagnosis may be difficult because of various clinical symptoms. In addition, patients with inflammatory diseases should be dealt with caution because they are less responsive to conservative treatment and may develop chronic IGB more easily.

## References

[1] Butcher JD, Salzman KL, Lillegard WA. (1996). Lower extremity bursitis. Am Fam Physician..

[2] Chafetz N, Genant HK, Hoaglund FT. (1982). Ischiogluteal tuberculous bursitis with progressive bony destruction. J Can Assoc Radiol..

[3] Larsson LG, Baum J (1986). The syndromes of bursitis. Bull Rrheum Dis..

[4] Le Floch P. (1982). [Serous ischial bursa]. Bull Assoc Anat (Nancy)..

[5] Swartout R, Compere EL (1974). Ischiogluteal bursitis. The pain in the arse.. JAMA..

[6] Rask MR (1980). "Snapping bottom": subluxation of the tendon of the long head of the biceps femoris muscle. Muscle Nerve..

[7] Fujisawa Y, Ito M, Nakamura Y, Furuta J, Ishii Y, Kawachi Y (2010). Perforated ischiogluteal bursitis mimicking a gluteal decubitus ulcer in patients with spinal cord injury: report of 2 cases. Arch Dermatol..

[8] Schuh A, Narayan CT, Schuh R, Honle W (2011). Calcifying Bursitis ischioglutealis: *>A Case report.*. J Orthop Case Reports..

[9] Lee YB, Kim DH, Jung JH, Park JY. (2017). Chronic Open Infective Lateral Malleolus Bursitis Management Using Local Rotational Flap. Biomed Res Int..

[10] Pompan DC (2016). Pes anserine bursitis: an underdiagnosed cause of knee pain in overweight women. Am Fam Physician..

[11] Nurkovic J, Jovasevic L, Konicanin A, Bajin Z, Ilic KP, Grbovic V (2016). Treatment of trochanteric bursitis: our experience. J Phys Ther Sci..

[12] Baumbach SF, Lobo CM, Badyine I, Mutschler W, Kanz KG (2014). Prepatellar and olecranon bursitis: literature review and development of a treatment algorithm. Arch Orthop Trauma Surg..

[13] Cho KH, Lee SM, Lee YH, Suh KJ, Kim SM, Shin MJ (2004). Non-infectious ischiogluteal bursitis: MRI findings. Korean J Radiol..

[14] Chen B, Rispoli L, Stitik T, Leong M (2017). Successful treatment of gluteal pain from obturator internus tendinitis and bursitis with ultrasound-guided injection. Am J Phys Med Rehabil..

[15] Cross GB, Moghaddas J, Buttery J, Ayoub S, Korman TM (2016). Don't aim too high: avoiding shoulder injury related to vaccine administration. Aust Fam Physician..

[16] Aaron DL, Patel A, Kayiaros S, Calfee R (2011). Four common types of bursitis: diagnosis and management. J Am Acad Orthop Surg..

[17] Reilly D, Kamineni S. (2016). Olecranon bursitis. J Shoulder Elbow Surg..

[18] Paluska SA (2005). An overview of hip injuries in running. Sports Med..

[19] Hitora T, Kawaguchi Y, Mori M, Imaizumi Y, Akisue T, Sasaki K (2009). Ischiogluteal bursitis: a report of three cases with MR findings. Rheumatol Int..

[20] Mills GM, Baethge BA (1993). Ischiogluteal bursitis in cancer patients: an infrequently recognized cause of pain. Am J Clin Oncol..

[21] Long SS, Surrey DE, Nazarian LN (2013). Sonography of greater trochanteric pain syndrome and the rarity of primary bursitis. AJR Am J Roentgenol..

[22] Akisue T, Yamamoto T, Marui T, Hitora T, Nagira K, Mihune Y (2003). Ischiogluteal bursitis: multimodality imaging findings. Clin Orthop Relat Res..

[23] Stell IM (1999). Management of acute bursitis: outcome study of a structured approach. J R Soc Med..

[24] Ernst E, Fialka V (1994). Ice freezes pain? A review of the clinical effectiveness of analgesic cold therapy. J Pain Symptom Manage..

[25] Acar N (2017). Low-energy versus middle-energy extracorporeal shockwave therapy for the treatment of snapping scapula bursitis. Pak J Med Scj..

[26] Khosrawi S, Taheri P, Ketabi M (2017). Investigating the effect of extracorporeal shock wave therapy on reducing chronic pain in patients with pes anserine bursitis: a randomized, clinical- controlled trial. Adv Biomed Res..

[27] Navarro-Zarza JE, Villasenor-Ovies P, Vargas A, Canoso JJ, Chiapas-Gasca K, Hernandez-Diaz C (2012). Clinical anatomy of the pelvis and hip. Reumatol Clin..

[28] Williams BS, Cohen SP (2009). Greater trochanteric pain syndrome: a review of anatomy, diagnosis and treatment. Anesth Analg..

[29] Johnson DB, Varacallo M (2019). Ischial Bursitis. StatPearls..

[30] Dierckman BD, Guanche CA. (2012). Endoscopic proximal hamstring repair and ischial bursectomy. Arthrosc Tech..

